# The impact of early surgical intervention in free intestinal perforation: a time-to-intervention pilot study

**DOI:** 10.1186/s13017-015-0047-0

**Published:** 2015-11-06

**Authors:** Andreas Hecker, E. Schneck, R. Röhrig, F. Roller, B. Hecker, J. Holler, C. Koch, M. Hecker, M. Reichert, C. Lichtenstern, G. A. Krombach, W. Padberg, M. A. Weigand

**Affiliations:** Department of General and Thoracic Surgery, University Hospital Giessen, Rudolf-Buchheim-Street 7, 35392 Giessen, Germany; Department of Anesthesiology, Surgical Intensive Care Medicine and Pain Therapy, University Hospital Giessen, Rudolf-Buchheim-Street 7, 35392 Giessen, Germany; Department of Radiology, University Hospital Giessen, Rudolf-Buchheim-Street 7, 35392 Giessen, Germany; Department of Internal Medicine, University Hospital Giessen, Rudolf-Buchheim-Street 7, 35392 Giessen, Germany

**Keywords:** Time-to-intervention, Free intestinal perforation, Sepsis

## Abstract

**Purposes:**

An abdominal inflammatory focus is the second most often source of sepsis with a high risk of death in surgical intensive care units. By establishing evidence-based bundled strategies the surviving sepsis campaign provided an optimized rapid and continuous treatment of these emergency patients. Hereby the hospital mortality decreased from 35 to 30 %. Sepsis treatment is based on three major therapeutic elements: surgical treatment (source control), antiinfective treatment, and supportive care. The international guidelines of the surviving sepsis campaign were updated recently and recommend rapid diagnosis of the infection and source control within the first 12 h after the diagnosis (grade 1c). Interestingly this recommendation is mainly based on studies on soft tissue infections.

**Methods:**

In this retrospective analysis 76 septic patients with an intraabdominal inflammatory focus were included. All patients underwent surgery at different time-points after diagnosis.

**Results:**

With 80 % patients of the early intervention group had an improved overall survival (vs. 73 % in the late intervention group).

**Conclusions:**

Literature on the time dependency of early source control is rare and in part contradicting. Results of this pilot study reveal that immediate surgical intervention might be of advantage for septic emergency patients. Further multi-center approaches will be necessary to evaluate, whether the TTI has any impact on the outcome of septic patients with intestinal perforation.

## Introduction

Despite modern diagnostic and therapeutic developments the in-hospital mortality of septic patients still remains inacceptably high. With 60 % mortality rates in cases of severe sepsis and septic shock a continuous optimization of treatment and rapid diagnosis is necessary and life-saving.

In up to 25 % of all septic patients an intraabdominal inflammation can be detected [1]. Results of the PROWESS study reveal, that in 66,5 % of the surgical patients the peritoneal cavity was affected by an inflammatory focus [2]. On a surgical intensive care unit the secondary peritonitis due to an intestinal perforation or an anastomotic leakage with extraintestinal air in the radiographic imaging is the main cause for an intraabdominal sepsis and sepsis-associated death. In a post mortem analysis of 235 patients, who died on surgical intensive care units, a persistent, continuous septic intraabdominal focus was found in 32,5 % of cases [1]. Compared to patients from medical intensive care units septic patients from surgical intensive care units have a 7 day longer length of hospital stay and higher cost rates [3].

While an intestinal perforation on its own leads to a mortality of about 14 %, a septic clinical progress is associated with an increase in mortality to 30 %. Among the patients suffering from secondary peritonitis the postoperative, secondary peritonitis bears the highest risk of a lethal outcome (1-year mortality >70 %) [4].

In February 2013 the new Surviving Sepsis Guideline was published, which underlines the multimodal, rapid treatment for the septic patient with an intraabdominal focus [5]. According to the recent guideline the sepsis therapy can be subdivided into four different subtypes: surgical source control, the antiinfectious therapy, the supportive intensive care medicine and adjunctive therapeutic approaches.

Surgical approaches for the treatment of intraabdominal infections are mainly based on principle and tradition. Over the last decade evidence-based medicine has emerged to assure best clinical practice, based on a review of literature, quality of research and therapeutic impact. Only few surgical strategies have been evaluated by randomized, prospective and controlled trials. Intraoperative circumstances are often unique and require a flexible, somtimes even unstandardized reaction by the surgeon.

Surgical source control is the only causal treatment option for patients suffering from peritonitis and intraabdominal sepsis. If source control is not possible during the initial emergency operation, mortality increases from 13 to 27 % [6].

In 2004 Barie et al. showed that an inadequate surgical removal of the intraabdominal inflammatory focus leads to a mortality of more than 90 % [7]!

The recent Surviving Sepsis Guideline from 2013 recommends an early source control within 12 h after diagnosis (evidence grade 1C) [5]:

*“A specific anatomical diagnosis of infection requiring consideration for emergent source control be sought and diagnosed or excluded as rapidly as possible, and intervention be undertaken for source control within the first 12 hr after the diagnosis is made, if feasible.”*

While a time-dependency in the early phase of hospital admission has been shown for an antimicrobial treatment at least in some studies, it remains nebulous, if a time-dependency of surgical source control and the outcome of the septic patient could be detected. These effects have never been analyzed before for the surgical patient with intestinal perforation. Interestingly, the guideline recommendation is based on literature mainly dealing with necrotisizing soft tissue infections [8–10]. Furthermore, the necrosectomy of peripancreatic necrosis is citated as one rationale for a conservative, delayed surgical intervention [11, 12]. To our surprise there is hardly any literature, analyzing the influence of the time to intervention on the outcome of critically ill septic patients with an intestinal perforation. For that reason we performed this time-to-intervention pilot study to investigate, if surgical source control in the very early phase of early-goal directed sepsis therapy is of benefit for our surgical intensive care patients.

## Materials and methods

**Fig. 1:**
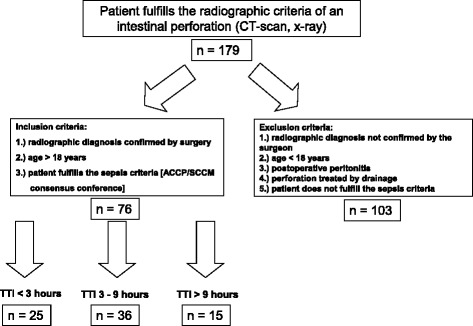
Study protocoll of this retrospective analysis of patients with an intestinal perforation at our university hospital: Of 179 patients with the diagnosis of a perforation in the GI-tract 79 met the inclusion criteria of this pilot study. These patients were attributed to the three study arms according to their time-to-intervention

This study was approved by the local ethical committee and was designed as a retrospective cohort analysis of clinical data from 76 septic patients (45 males and 31 females) with a mean age of 59.64 years (range 21–88 years) suffering from intestinal perforation. Observational period was between August 2008 and February 2012. Subjects were included, if intestinal perforation was detected by radiographic imaging (computertomography scan or x-ray of the abdomen) and confirmed during surgery. Intestinal perforation was diagnosed in cases of free intraabdominal air and/or extravasation of contrast medium into the peritoneal cavity. Patients were included, if they showed either free intestinal perforation or an anastomotic leakage. They were excluded, if the intraoperative and radiologic findings were discordant, of if there was a contained perforation. In addition, patients must have met the sepsis criteria of the ACCP/SCCM consensus conference on sepsis [13] [Fig. 1].

Patients were excluded from the study if the radiological diagnosis of an intestinal perforation was discordant to the intraoperative finding. Further exclusion criteria were postoperative perforations due to anastomotic leakage, age < 18 years and perforations, which were treated interventionally (e.g. CT-guided drainage, Endo-VAC-Systems). Patients with an open abdomen were furthermore excluded from the study.

“Time-to-intervention” is defined as the time span between the diagnosis by radiographic imaging (CT scan/x-ray) and the start of surgical intervention (“cut of the scin”). Due to the time to intervention the study group was subdivided into three groups: under 3 h, 3 to 9 h and longer than 9 h. These time intervals were chosen in order to represent usual hospital procedures. In daily routine a time to intervention of less than 3 h reflects the situation, when the surgeon is waiting for the next operation room available (high urgency). Patients who were operated between 3 and 9 h represent urgent cases, which were operated within one hospital working shift. More than 9 h of time to intervention represent a situation of a delayed diagnosis and/or treatment of the intestinal perforation.

Clinical data were collected by the regular hospital documentation software (IMESO KisData and ICUData version 7.7.0.1590, KAOS desktop version 3.0.0.1, MEDOS Nexus .med RIS Client version 9.3.2276). Parameters of interest were length of hospital stay, ventilatory time, pre- and postoperative blood parameters, amount of cristalloids and catecholamine consumption. Furthermore, the application of blood transfusions was analyzed. Different protein serum levels (lactate, pH, base excess, blood glucose, creatinine, urea, c-reactive protein, procalcitonin), hemoglobin and the amount of leukocytes were measured on admission in the emergency room and immediately after surgical source control. Both the in-hospital mortality and the overall motality were calculated. Additionally radiologic methods and surgical specifications (cause and location of perforation, surgical technique) were collected. The amount and type of infusion and transfusion were determined for the first 24 h after surgery. ICU prognostic scores (SAPS II, SOFA, APACHE II) were also calculated for the first postoperative 24 h according to the established protocols published before [14–16].

Baseline characteristics were expressed as mean ± standard deviation in normal and as median ± interquantile range in not normally distributed data. A *p*-value of *p* < 0.05 was considered as significant. Descriptive analysis was performed with contingency tables, while Chi-Squared-Test was used to describe the distribution of discrete parameters in proportion to the time to intervention. Mann–Whitney-U-Test was used to test metric values. All statistical analyses were performed with Microsoft^**®**^ Excel^**®**^ and IBM^**®**^SPSS^**®**^Statistics (version 21.0.0.0).

## Results

**Fig. 2:**
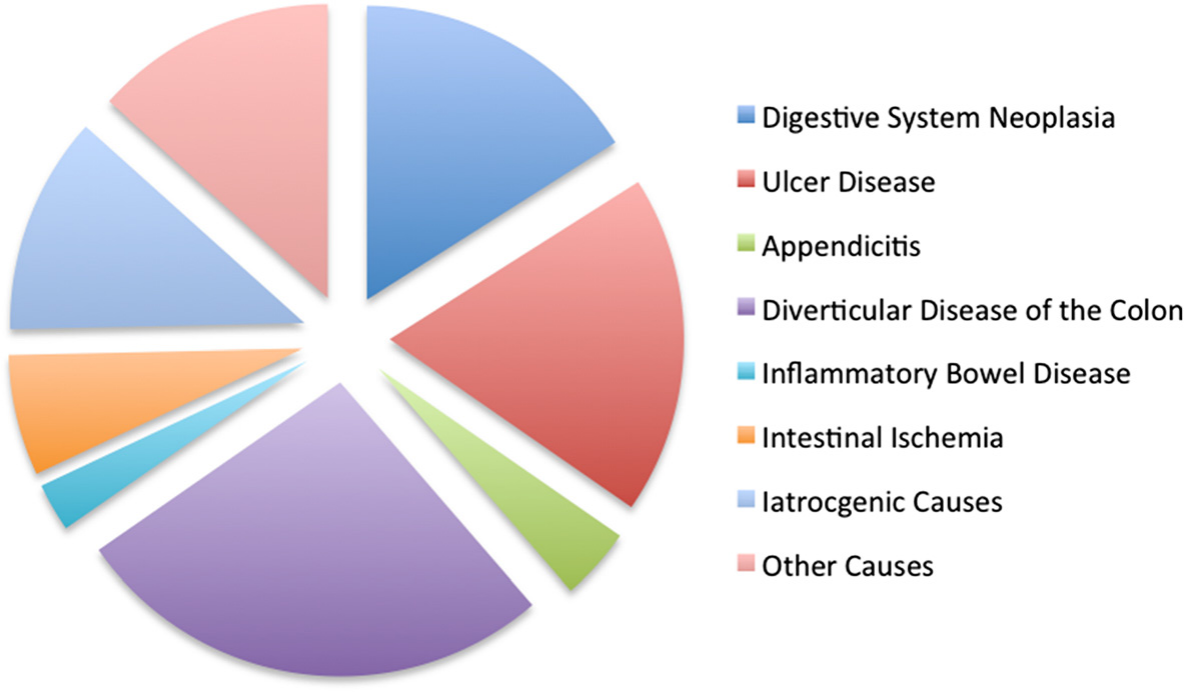
The different reasons for the perforation reflects the typical pattern of a European university hospital

From 179 patients with radiographic signs of an intestinal perforation 76 septic patients fulfilled the inclusion criteria. 31 female and 45 male septic patients with an intestinal perforation entered this retrospective monocenter study. Figure 2 shows the location of the intraabdominal inflammatory. With 36 cases most patients were operated between 180 and 540 min after diagnosis (study group II). In 25 patients surgery was performed in less than 180 min (study group I). In study group III (TTI > 540 min) 15 cases were included.

In the majority of cases (97 %) the diagnosis was based on an emergency computertomography scan. In two cases free intraabdominal air in the conventional x-ray of the abdomen led to the indication for surgical intervention.

**Fig. 3:**
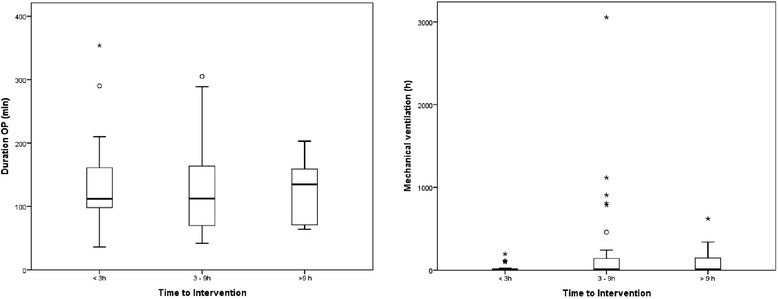
All study groups were equal concerning the duration of surgical source control. The time-to-intervention had no significant impact on the time of mechanical ventilation or the amount of catecholamins adiministered after surgery. o and * represent single spike values

Sixty patients developed respiratory failure requiring mechanical ventilation in the postoperative course. The duration of the operation did not differ between the three groups. In addition, the absolute time of mechanical ventilation and the amount of catecholamines applied did not depend on the time between diagnosis and surgical intervention (Fig. 3). Revisions had to be performed in 38 % of the cases (study group 1 44 % vs. study group 2 39 % vs. study group 3 27 %). In case of perforation of the small intestine segmental resection with a side-to-side anastomosis were performed. Perforations of the sigmoid colon were resected. Due to the impression of the emergency surgeon a Hartmann operation was done. Alternatively the colorectum was reconstructed by a descendorectostomy. In some cases (deep distal anastomosis) a diverting protective ileostomy was implemented.

Perforations of the ascending colon were reconstructed by a ileotransversostomy. In cases of anastomotic leakage either discontinuous or continuous reconstructions were performed due to the surgeon’s impression.

Concerning the 30-day in hospital mortality (non-significant) differences between the three study groups were detected: While patients of study group I had a 80 % survival, patients with a time-to-intervention of more than three hours (study group II and III) showed a survival of 73 % each. The overall survival was 80 % for the early intervention group, compared to 75 % (study group II) and 73 % (study group) respectively.

Tables 1, 2 and 3 provide the descriptive parameters, retrospectively analyzed in this study.

**Table 1:** Characteristics of the three study groups

			<3 h			3–9 h				>9 h				
	*N*	*n*	Mean	SD	Median [IQR]	*n*	Mean	SD	Median [IQR]	*n*	Mean	SD	Median [IQR]	*p*-Value
Age	76	25	59,64	18,775	63 [50, 74]	36	64,36	14,934	65 [56.25;76.5]	15	68,8	14,586	69 [61;80]	0,205
Length of hospital stay	76	25	20,64	14,731	22 [10, 28]	36	28,75	32,056	17,5 [11;29]	15	20,27	14,235	16 [11;27]	0,921
Duration of surgery (minutes)	76	25	135,2	73,005	112 [97, 165.5]	36	126,58	68,393	112,5 [70;170.25]	15	127,07	47,596	135 [70; 160]	0,744
Ventilatory time (hours)	76	25	29,524	51,3765	6,2 [0.5, 19.2]	36	228,253		9,9 [5.175; 148.75]	15	105,94	186,3957	7,7 [3.9;276]	0,141
Katecholamins (within 24 h)	76	25	7102,32	15541,449	450 [0, 9330]	36	31294,5	80932,459	1911 [0; 9700]	15	4570,8	8733,606	0 [0; 2340]	0,604
Bikarbonate	76	25	84	123,929	0 [0, 155]	36	65,56	91,634	50 [0; 100]	15	40	54,116	0 [0;100]	0,656
Kristalloids	76	25	2876	1416,945	2600 [1600, 4125]	36	2564,17	1364,581	2525 [1562.5; 31^37.5]	15	1990	978,373	1800 [1500; 2100]	0,184
Kolloids	76	25	854	1951,384	500 [0, 1000]	36	326,39	421,813	0 [0; 500]	15	400	430,946	500 [0; 500]	0,13
Substitution of erythrocyte concentrates	76	25	276	465,725	0 [0, 1020]	36	283,33	411,617	0 [0; 990]	15	260	0	437,199 [0; 1200]	0,839
Thrombocyte concentrates	76	25	20	70,711	0	36	20,83	71,088	0	15	33,33	129,099	0	1
Fresh frozen plasma	76	25	100	322,749	0	36	187,5	432,497	0	15	133,33	296,808	0	0,322
Glucose substitution within24h	76	25	15,2	76	0	36	15,2	76	0	15	40	129,835	0	0,987
Bicarbonate substitution within 24 h	76	25	70	119,024	0 [0, 100]	36	48,53	101,865	0	15	53,33	104,312	0 [0; 50]	0,335
Kistralloid substitution within 24 h	76	25	2990,8	2348,709	2800 [1720, 3700]	36	3198,61	1712,078	2800 [2000; 4500]	15	2536,67	1155,649	2250 [1700; 3000]	0,79
Kolloid substitution within 24 h	76	25	380	505,8	0 [0, 500]	36	328,57	404,076	0 [0; 500]	15	833,33	1531,417	0 [0; 1000]	0,869
Erythrocyte concentrates within 24 h	76	25	108	285,657	0	36	233,33	424,937	0 [0; 300]	15	260	337,639	0 [0; 600]	0,056
Thrombocyte concentrates within 24 h	76	25	12	60	0	36	12	60	0	15	8,33	50	0	0,604
FFP within 24 h	76	25	48	132,665	0	36	177,78	390,685	0 [0; 350]	15	26,67	103,28	0	0,283
SAPS_II score after source control	76	25	42,36	15,508	43 [29.5, 50.5]	36	44,28	15,161	46 [31.25; 55.75]	15	41,53	13,674	38 [34; 46]	0,894
APACHE II score after soruce control	76	25	21,08	8,026	21 [14, 26]	36	21,86	6,69	21,5 [17.25; 26.5]	15	19,87	5,718	20 [16; 25]	0,79
SOFA score after source control	76	25	6,32	4,059	6 [3, 9.5]	36	7,28	3,377	7 [5; 10]	15	6,27	3,127	6 [4; 9]	0,479
Gastric reflux within 24 h (ml)	76	25	166	225,074	30 [0, 300]	36	266,94	436,347	100 [0; 400]	15	163,33	227,272	80 [0; 250]	0,57

*FFP* fresh frozen plasma, *SAPS* Simplified Acute Physiology Score, *APACHE* Acute Physiology and Chronic Health Evaluation II-score, *SOFA* Sequential Organ Failure Assessment score

**Table 2:** Descriptive analysis of different parameters of the three study groups

		<3 h				3–9 h				>9 h				
	*N*	*n*	Mean	SD	Median [IQR]	*n*	Mean	SD	Median [IQR]	*n*	Mean	SD	Median [IQR]	*p*-Value
Before surgery														
Serum lactate	46	9	2,45	1,66193	1,9 [1.2, 3.2]	28	2,6404	2,67295	1,8 [1.175; 2.875]	9	1,9733	0,99991	1,7 [1.235; 2.645]	0,661
pH	49	17	7,34	0,05374	7,33 [7.3, 7.375]	23	7,3422	0,10904	7,38 [7.24; 7.41]	9	7,4144	0,04613	7,43 [7.4; 7.445]	0,074
BE	49	17	−3,3824	2,56131	−3,8 [−5.35, −0.9]	23	−4,8	6,70271	−3,8 [−8.5; 0.4]	9	−1,0778	6,41693	−2,2 [−5.4; 1.4]	0,95
Glucose	55	11	152,684	70,4683	138 [101, 166]	33	150,494	73,9948	136 [100; 165]	11	163,909	68,9775	148 [107; 176]	0,869
Hemoglobin	75	24	12,417	3,0039	12,6 [9.7, 14.65]	36	13,681	13,8924	11 [9.475; 13.575]	15	10,593	1,9036	9,9 [9.1; 11.8]	0,11
Creatinine	76	25	1,352	1,138	1 [0.8, 1.25]	36	7,136	34,2748	1 [0.8; 2.05]	15	1,347	0,9782	1 [0.8; 1.5]	0,781
Urea	76	25	59,68	54,4225	41 [25, 83]	36	64,764	62,7524	42,5 [32.25; 73.75]	15	76,133	41,8857	63 [43; 108]	0,175
Leukocytes	76	25	12,276	7,2799	12,3 [7.25, 17.2]	36	12,328	8,8553	9,25 [7.975; 15.775]	15	11,373	7,0199	9,3 [6.1; 12.9]	0,808
CRP	75	25	141,6148	157,91994	74,3 [9.05, 253]	36	168,0072	131,14877	149,175 [42.32; 250.28]	14	181,7514	137,22675	155,7 [56.28; 320.4]	0,176
PCT	12	3	13,267	14,584	10,6 [0.2, 0	7	8,714	12,3999	0,8 [0.4; 15]	2	12,95	15,3442	12,95 [2.1; 0]	1
After Surgery														
Serum lactate	66	15	2,9718	2,19183	2,25 [1.35, 4]	36	1,9333	1,41219	1,4 [1.1; 2.775]	15	2,3867	1,92423	1,8 [1.2; 2.2]	0,06
pH	74	23	7,302	0,082848	7,308 [7.231, 7.37]	36	7,346	0,079749	7,35 [7.31; 7.4]	15	7,33233	0,080053	7,31 [7.265; 7.4]	0,072
BE	74	23	−5,4348	3,49488	−5,1 [−8.4, −3]	36	−3,7389	3,98929	−3,8 [−5.4; −2.2]	15	−3,8467	5,18871	−4,5 [−6.2; −2.3]	0,059
Glucose	75	24	126,288	42,1621	122 [1104, 145.75]	36	130,717	51,9907	134,5 [107.25; 144.75]	15	172,467	54,1702	175 [138; 221]	0,118
Hemoglobin	75	24	16,438	22,6004	11,9 [10.25, 13.68]	36	17,181	28,0558	10,65 [9.425; 13.08]	15	10,367	2,4555	10,4 [9.6; 11.6]	0,065
Creatinine	75	24	1,304	1,1035	1 [0.9, 1.12]	36	1,45	0,7865	1,2 [0.825; 1.7]	15	1,267	0,8156	0,9 [0.8; 1.6]	0,319
Urea	75	24	54,67	47,416	39,5 [26.25, 49.5]	36	71,08	56,263	59 [36; 78]	15	74,13	43,083	67 [47; 83]	0,025
Leukocytes	75	24	10,129	5,9572	9,4 [4.95, 13.4	36	10,597	6,5088	8,45 [6.43; 13.2]	15	12,02	7,4694	12,9 [4.6; 16.9]	0,759
CRP	75	24	137,0608	110,5366	140,93 [31.2, 229.4]	36	168,595	107,2545	156,555 [76.8; 228.5]	15	182,9213	104,25169	184,2 [115; 256]	0,175
PCT	13	3	20,933	20,9381	17,7 [1.8, 0]	9	20,933	20,9381	17,7 [5.35; 29.6]	1	26,356	34,8009	14,4 [9.75; 30.5]	1

*BE* base excess, *CRP* C-reactive protein, *PCT* procalcitonin

**Table 3:** General description of the three study groups conerning OP technique, antibiotics and others

	Total		Up to 3 h.		3 to 9 h.		More than 9 h.	
Gender	n	%	n	%	n	%	n	%
Female	31	41 %	8	32 %	13	36 %	10	67 %
Male	45	59 %	17	68 %	23	64 %	5	33 %
Total	76	100 %	25	100 %	36	100 %	15	100 %
Ventilation	N	%	n	%	n	%	n	%
Not ventilated	16	21 %	5	20 %	8	22 %	3	20 %
Ventilated	60	79 %	20	80 %	28	78 %	12	80 %
Total	76	100 %	25	100 %	36	100 %	15	100 %
OP-method	N	%	n	%	n	%	n	%
Laparascopic	2	3 %	2	8 %	0	0 %	0	0 %
Open	74	97 %	24	92 %	35	97 %	15	100 %
Converted	2	3 %	0	0 %	1	3 %	0	0 %
Total	76	100 %	26	100 %	36	100 %	15	100 %
Antibiotics	N	%	n	%	n	%	n	%
No antibiotics	2	3 %	0	0 %	0	0 %	2	13 %
Antibiotics	74	97 %	25	100 %	36	100 %	13	87 %
Total	76	100 %	25	100 %	36	100 %	15	100 %
Revision	N	%	n	%	n	%	n	%
No revision	47	62 %	14	56 %	22	61 %	11	73 %
Revision	29	38 %	11	44 %	14	39 %	4	27 %
Total	76	100 %	25	100 %	36	100 %	15	100 %
Ischemia	N	%	n	%	n	%	n	%
No ischemia	67	88 %	22	88 %	31	86 %	14	93 %
Ischemia	9	12 %	3	12 %	5	14 %	1	7 %
Total	76	100 %	25	100 %	36	100 %	15	100 %

**Fig. 4:**
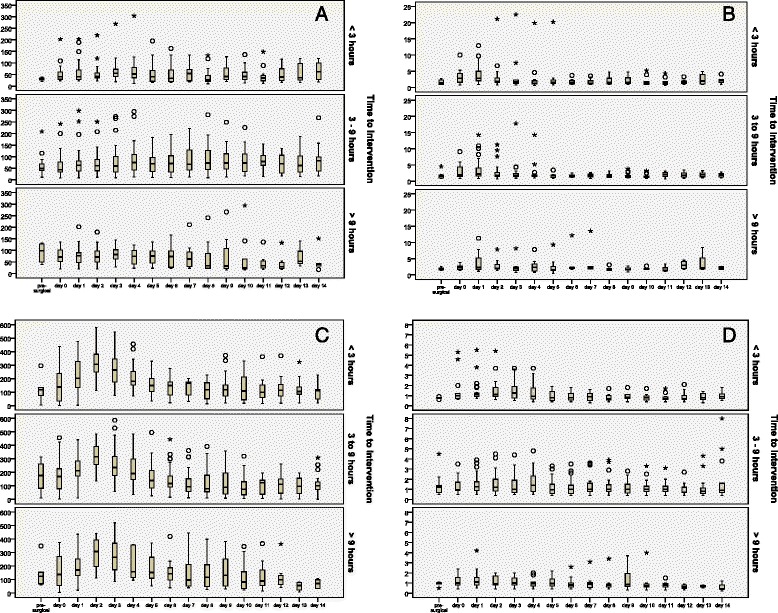
The time-courses of creatinine (**a**), urea (**b**), C-reactive protein (CRP) (**c**) and serum lactate (**d**) before surgical source control and in the postoperative phase. o and * represent single spike values

The time-courses of the serum CRP, urea, creatinine and lactate are presented in Fig. 4. Significant differences between the three study groups could not be detected.

## Discussion

Despite the risks of a specific surgical intervention (fistulas, SIRS, bleeding) it is common surgical practice, that emergency patients with intestinal perforation are initially stabilized and transferred to the operation room.

In our pilot study 76 septic patients were analyzed concerning the time-dependency between surgical intervention and patient’s mortality and morbidity. It thus provides insights into the surgical management in the very early phase after hospital admission:

More than 80 % of the patients with an intestinal perforation underwent surgical source control within the first 9 h after hospital admission. About one third of the patients was operated within 3 h. In the great majority of cases we met the 12-h intervall of surgical source control strongly recommended by the Surviving Sepsis Campaign [5]. The overall survival was 80 % for study group I and decreased to 75 % (study group II) and 73 % (study group III) respectively.

In a multivariate analysis of septic patients with necrotizing soft tissue infections Boyer et al. was able to show that a delayed surgical intervention > 14 h after diagnosis was a negative prognostic predictor, associated with a 34-fold increase in hospital mortality [9]. Hospital mortality was 40.6 %. Forty-one percent of the patients were septic on admission.

Delayed operation is recognized as a contributor to adverse outcome in many fields of emergency surgery. A prospective trial from Kollef could determine the delay in diagnostics followed by surgical intervention as one independent negative predictor for the survival of intensive care patients [17]. Reasons for delayed surgical intervention were analyzed in a retrospective study by North et al. and by the Danish National Indicator Project in 2009: out-hospital perforation, masked clinical signs of peritonitis, late surgical attendance and a missing pulseoxymetry on admission were independent prognostic factors for a bad outcome.

In a study from the field of pediatric surgery all children, who survived a necrotizing fasciitis had underwent surgical source control within three hours after admission [8]. These data are supported by a retrospective cohort study by Gajic et al., who could identify delays in surgical evaluation and therapy as critical contributors to mortality on medical intensive care units: A delay of more than 48 h led to a dramatic decline in patients’ survival (41 % vs. 73 % in the early intervention group) [18].

Despite some evidence for a conservative treatment of perforated peptic ulcera [19] surgery is still the gold standard for gastric perforation [20]. For patients with perforations of the upper GI tract Buck et al. could detect a strong dependency of the delay of surgical treatment after hospital admission on the 30 day survival rates [21]. Over the first 24 h after admission each hour of surgical delay was associated with a 2 % decrease in patient survival [21]. The amount of septic patients in the study group remains unclear. About 16 % were hypotensive on admission.

This strong time-dependency conflicts the recommendation of the new guidelines of the Surviving Sepsis Campaign, which advises a time-to-intervention of 12 h from diagnosis to source control. These very early hours after hospital admission are still nebulous concerning the influence of the velocity of diagnostics and surgical source control on patients’survival.

Due to the very limited number of septic patients included in this pilot study we potentially failed to find a significant influence of the TTI on the mortality. Nevertheless with prolonged TTI mortality rates showed an increasing tendency to higher levels. While 30-day mortality was 20 % in Study group I, it was 27 % in group III. Hospital mortality confirmed this tendency (study group I 20 % vs. Study group II 33 % vs. Study group III 33 %).

In contrast to Wachta et al., who investigated 14 % mortality for patients with peritonitis after intestinal perforation, our study revealed a higher overall mortality (31 %). This can be explained by the inclusion of only septic patients with increased APACHE II scores > 20 (vs. APACHE II 14.3). Besides the lack of prospective randomized studies a comparison of studies on surgical source control ist nearly impossible. This is due to incoherence in the group of patients with intestinal perforation: While patients with perforations requiring intensive care have a very poor prognosis, patients with iatrogenic perforation show an improved survival [18, 22]. Perforations due to an ischemia of the bowel have a very bad prognosis. With a 1 year-mortality of more than 70 % patients with secondary peritonitis due to postoperative complications have the worst outcome [4]. Generally peritonitis is a negative predictor for patients’ outcome after intestinal perforation. In our study an early surgical intervention tends to result in lower rates of peritonitis (group I 88 % vs. Group II 92 % vs. Group III 100 %). This goes in line with the differences in mortality we could determine.

To minimize the incoherence of the study group the patients with complications after surgery like anastomotic leakage or ischemia were excluded from the study cohort. Nevertheless this important group of patients for the surgeons everyday-life should be analyzed in future studies.

This incoherence in the group of septic patients in general also reflects the contradictory results on the impact of a rapid or delayed initiation of antimicrobial therapy on the mortality of septic patients: The prospective, objective multicenter study from Ferrer et al. revealed, that only an early broad-spectrum antibiotic treatment within one hour is life-saving for the septic patient [23]. However, source control was not tested in this study. These data underline the results of Kumar et al., who determined the impact of delays in initiation of an effective antibiotic treatment on the mortality of septic shock patients. Each hour of delay in the adminitration of antimicrobials was associated with a decreased survival of 5–10 % [24]. Despite its wide adoption, whether this practice is of benefit for all septic patients is still controversial. Hranjec et al. analyzed 1483 patients concerning an aggressive (early start of antimcirobial treatment) vs. conservative (start of antibiotics as soon as the infection objectively was confirmed) treatment modality. Interestingly, the benefitial effects of an early administration of antibiotics were not detectable in the hemodynamic stable surgical patient [25]. Detailed analysis of the study protocols reveals that the beneficial effects of a rapid initiation of antimicrobial pharmacotherapy especially can be found for the subgroup of patients with a septic shock, a group, which also received antibiotics in the Hranjec study immediately.

From these data one could deduce that not the standardized, strictly time-dependent, but the personalized antimicrobial therapy could be trend-setting.

The originality of our study is supported by several differences to trials published previously: First only those patients were included, who met the sepsis criteria of the ACCP/SCCM consensus conference on sepsis. Second, all patients had an intestinal perforation. The locations of the perforations and its reasons reflect the spectrum of a typical European university hospital. Third, all study groups showed no differences concerning their catecholamine consumption or APACHE II scores. Thus the groups are comparable concerning the severity of sepsis.

This small unicenter study is a pilot-study to turn the focus on citically ill, septic surgical patients, which – so far – are not reflected by the recent sepsis guidelines.

Due to the retrospective study design the analysis of the patients suffering from severe abdominal sepsis bears the risk of an important bias: It is probable, that those patients, who were staged as very critical in the emergency room were transferred into the operation room more rapidly than those, who presented as relatively stable concering their clinical situation. We tried to minimalize this bias by analysing the consumption of catecholamines, the analysis of preoperative labarotory parameters and clinical scores (e.g. APACHE II). Our study was designed and performed in only one surgical center and thus only a very limited number of septic patients were included. Results can only show tendencies, which underlines, that multicenter approaches are necessary to analyze this group of critically ill surgical patients in the very early phase of emergency and intensive care treatment.

## Conclusions

Despite its relevance literature on the time dependency of early surgical intervention in intestinal perforation is rare and contradictory. In this pilot study there is a tendency that immediate surgical intervention might be of advantage for septic emergency patients. Further multi-center approaches will be necessary to evaluate, whether the TTI has any impact on the outcome of septic patients with intestinal perforation.

## Abbreviations

APACHE: acute physiology and chronic health evaluation
SAPS: simplified acute physiology score II
SOFA: sepsis-related organ failure assessment
TTI: time to intervention
